# Community Engaged Lifestyle Modification Research: Engaging Diabetic and Prediabetic African American Women in Community-Based Interventions

**DOI:** 10.1155/2016/3609289

**Published:** 2016-07-14

**Authors:** Starla Hairston Blanks, Henrie Treadwell, Anya Bazzell, Whitney Graves, Olivia Osaji, Juanita Dean, James T. McLawhorn, Jareese Lee Stroud

**Affiliations:** ^1^Morehouse School of Medicine, Community Voices: Healthcare for the Underserved, 720 Westview Drive SW, Atlanta, GA 30310, USA; ^2^Morehouse School of Medicine, Department of Community Health and Preventive Medicine, 720 Westview Drive SW, Atlanta, GA 30310, USA; ^3^Columbia Urban League, 1400 Barnwell Street, Columbia, SC 29201, USA

## Abstract

*Purpose*. The I Am Woman (IAW) Program is a community-based, culturally responsive, and gender-specific nutrition, obesity, and diabetes educational prevention program designed for African American women (AAW). Chronic nutrition-related health conditions such as excess body weight, diabetes mellitus, cardiovascular disease, and some forms of cancer are common among many African American women.* Methods*. IAW engaged AAW at risk for such deleterious health conditions by developing a health education intervention that aimed to support weight loss and management, improve knowledge about healthy lifestyle behavioral choices, and facilitate increased access to comprehensive healthcare. This Community Health Worker- (CHW-) led program enrolled 79 AAW aged 18 and older in a 7-week group health education intervention.* Results*. Following the intervention, results indicated that participants had greater knowledge about nutrition and health, strategies for prevention and management of obesity and diabetes, increased engagement in exercise and fitness activities, and decreased blood pressure, weight, body, and mass index. Cholesterol levels remained relatively unchanged. Additionally, AAW visited a primary care doctor more frequently and indicated greater interest in addressing their health concerns.* Conclusion*. This model of prevention appears to be a promising approach for increasing awareness about ways to improve the health and well-being of AAW.

## 1. Introduction

The U.S. Department of Health and Human Services Office on Minority Health estimated that, in 2012, African Americans, including those of more than one race, comprised 15.2% of the total U.S. population [1]. The African American community bears a disproportionally high burden of major nutrition-related chronic diseases [2, 3] with 48% of adults suffering from a chronic disease relative to only 39% of the general population [3]. Common among African American women, these conditions—obesity, type 2 diabetes mellitus (diabetes), cardiovascular disease (primarily hypertension, heart disease, and stroke), and certain forms of cancer—are costly and are associated with substantial morbidity, mortality [2, 4, 5], and a reduced quality of life [4, 5]. Diet and nutrition are critical factors in the prevention and control of these diseases and, therefore, play an important role in nutrition-related interventions [2, 5–7]. It is imperative that complex lifestyle factors that include physical activity along with diet and nutrition be identified as important determinants of health [2, 6].

Obesity, a prominent health concern in the United States [2, 8, 9], is a risk factor for a myriad of chronic diseases [2, 6, 8, 9]. A study of obesity (body mass index (BMI) ≥ 30 kg/(m^2^) among American adults revealed that among all racial and ethnic groups, non-Hispanic African Americans had the highest overall obesity prevalence rate (47.8%) while non-Hispanic whites had the lowest (32.6%) rates (non-Hispanic Asians 10.8%). Furthermore, approximately 82% of African American women were overweight (BMI mass index 25.0–29.9) (kg/m^2^) or obese [10, 11], which represented the highest overweight and obesity combined rate among the women in all groups. Almost 13% (13.2%) of non-Hispanic African Americans 20 years of age or older were diagnosed with diabetes relative to non-Hispanic white adults [12]; and they had a 77% higher risk of diagnosed diabetes [13]. Additionally, women, compared to men, have a higher risk of diabetes [4] and the death rate for African Americans with this disease is more than twice that for whites [2]. These findings suggest that non-Hispanic African American women are at high risk for health conditions and health issues related to overweight and obesity.

African Americans are also disparately impacted by heart disease as well as other health conditions. While heart disease is the leading cause of death for all racial and ethnic groups in the United States [2, 4]; the mortality rate for this disease among African Americans is 1.3 times that of whites [2, 14]. Compared to women of other racial and ethnic groups in the United States, African American women are substantially more likely to have hypertension and to die from a stroke [15]. For most cancers, African Americans also have the highest mortality rate and lowest survival rate of any racial and ethnic group in the United States [2]. Due, at least in large part, to these health disparities, life expectancy at birth from 2009 to 2010 was significantly lower for African Americans than whites among men (71.8 versus 76.5 years) and women (78.0 versus 81.3 years) [16].

Americans are inundated with information, products, and services that promote the adoption of healthy dietary practices, in combination with regular physical activity. These practices aim to maintain a healthy body weight, reduce chronic disease risks, and control chronic illnesses. However, many Americans are not following dietary and physical activity recommendations despite the proven benefits of these strategies [2, 17].

The I Am Woman (IAW) Program sought to address the disconnect between nutrition-related knowledge, attitudes about healthy eating and exercise, and the adoption of behavior that support improved health. The IAW Program was designed to support African American women specifically at risk for obesity and diabetes. The program's emphasis on nutrition and physical activity reflects support for a highly targeted community-level strategy and intervention to assist African American women and their families in adopting and maintaining healthier lifestyle behavior. This program is a collaboration between General Mills Corporation; Columbia Urban League, an affiliate of the National Urban League; and Community Voices: Healthcare for the Underserved of Morehouse School of Medicine. The specific aims of IAW were to (1) increase participants' knowledge of nutrition, including healthy meal preparation and its impact on health; (2) improve select nutrition-related screening health indicators; and (3) increase the proportion of participants with a usual source and place of primary care through the establishment of community partnerships.

## 2. Materials and Methods

### 2.1. Project Setting

IAW was conducted in a community center in the metropolitan area of Richland County of Columbia, South Carolina. In 2010, Richland County had a population of 384,504, of which 45.9% were African American and 47.3% were white. In contrast, only 27.9% of the state population were African American and 66.2% were white. Richland County's median household income and per capita money income were $45,643 and $25,865 in 2009, respectively [18]. All clinical tests were performed at Eau Claire Medical Cooperative and were facilitated by registered nurses.

### 2.2. Project Participants

A convenience sample of 79 African American women that represented a diverse segment of residents from Richland County in Columbia, South Carolina, was included in the study. Women were eligible to participate if they were 18 years of age or older, were self-identified as African American, and were residents from the local community with a focus on the 29202 Columbia, SC zip code. A total of 136 potentially eligible women were recruited; however, 17 of these women became ineligible for the project because of preintervention health screening nonresponse, leaving a total of 119 who enrolled in the project. Forty of the 119 participants were subsequently excluded because they did not complete the project, yielding a final analysis sample of 79 and a completion rate of 66%.

### 2.3. Assessment Tools

All project participants completed a consent and baseline enrollment form, which captured demographic, educational, and insurance information. Participants also completed a preintervention questionnaire and a preintervention knowledge assessment and participated in health screenings for their weight, body mass index, blood pressure, blood cholesterol, and blood glucose levels before and after intervention conducted by a registered nurse.

### 2.4. Procedures

The Morehouse School of Medicine Institutional Review Board approved this study; and all project participants provided informed consent. Prior to implementation of the intervention, our research and evaluation team conducted preliminary activities to help inform the development of the educational model and curriculum. Specifically, two focus groups—each consisting of 14 women aged 23–64 and five key informant interviews with community health workers (CHWs), religious leaders, social service workers, and local healthcare system employees—were delineated. Focus group participants closely matched the sociodemographic and cultural characteristics of the study's target population. The feedback from women and key informants was used to establish and refine various components of the project particularly related to recruitment and retention strategies, the educational curriculum, study questionnaires, and physical activities (e.g., yoga, dance, and walking groups) that may encourage active and ongoing participation from women. This qualitative community informed strategy provided valuable insight into the utilization of recruitment strategies and the use of incentives that would not be coercive but viewed as a reward by potential participants.

Recruitment strategies included the use of word-of-mouth promotion, social media, a radio public service announcement, and local publication efforts (e.g., church newsletter announcements and flyers). Once an eligible woman agreed to participate, a CHW arranged the initial enrollment session. The CHWs gave participants details about the project and participants' expectations and responsibilities; and it was explained that participants would receive a variety of incentives over the course of the project (e.g., transportation vouchers, access to job placement resources, cash prizes, and gift cards). The incentive policy was reviewed and approved by the Institutional Review Board to ensure they would not be viewed as coercive for project participation and that they would be viewed as rewards for goal attainment. Incentives and prizes were valued between $5 and $25 with one $75 grand prize awarded to the participants who lost the greatest percentage of their body weight by the intervention conclusion. Participants were notified that there would be a “grand prize” but not of the exact amount until the final weigh-in.

To increase the feasibility of the project, a community-based participatory strategy was used, focused on a public health approach, to build capacity through partnerships and collaboration. Six CHWs from the targeted communities, who were high school graduates or had a general educational diploma (GED), were hired to work with IAW. They were required to attend a three-day training conducted by Community Voices: Healthcare for the Underserved staff and a consultant. Following this training, Community Voices staff followed up with the CHWs weekly to address outstanding issues and to ensure adherence to the educational protocol. In addition to training and monitoring the CHWs, Community Voices staff provided IAW with a myriad of evaluative, technical support, and awareness-building services. The CHWs were primarily responsible for recruiting participants, conducting the seven nutrition and fitness education sessions using a specified protocol, and supporting women with identifying a medical home. CHWs also helped participants to overcome barriers to accessing healthcare by assisting them in connecting to primary healthcare providers and other community resources.

### 2.5. Overview of the Educational Intervention

The IAW intervention, complemented with physical activity and implemented directly following the collection of the baseline data, included 3 primary components: (1) a culturally tailored, gender-specific nutrition education program in combination with physical activity that included 7 sessions (about 12 hours total); (2) group cardio and strength/resistance exercise sessions (6 hours total); and (3) connecting participants to a primary care physician/medical home and other community resources (e.g., food banks, parks, and other recreational facilities). The IAW curriculum was informed by best practices including the National Diabetes Education Program's Power to Prevent Diabetes curriculum [2, 7]. The curriculum includes the following sessions:

- Introduction to the healthy lifestyle program.
- Nutrition and chronic disease.
- Nutritional literacy and building nutritional competence.
- Combating stress and emotional eating.
- Strategies for health eating and exercise.
- Partnering with your healthcare provider.
- Celebrating your healthier family.

In addition to the sessions other resources and activities are a part of the program such as the following:

- Physical activity planning section.
- Healthy eating recipes.
- Separate youth focused curriculum which supplements the adult sessions.
- Community Resource Guide.
- Field trip/activity planner.

*Complimentary Kids Manual*. The participants received the IAW Women's Manual, which was used to guide the sessions, a Community Resource Guide, and project materials including portion measuring cups, a Food and Activity Tracker Form, and a pedometer.

The project began with a “kickoff cookout” designed to introduce residents to nutritious foods prepared in a healthy manner. Each nutrition session of the curriculum highlighted healthy meal and snack preparation for families. Additional key nutrition education opportunities included food preparation and cooking demonstrations, full-service grocery store visits, and a weekly assignment to prepare a low-fat or low-calorie recipe from the Betty Crocker Kitchen*™* or Betty Crocker Diabetes Cookbook*™*. The group exercise sessions provided an opportunity for participants to establish supportive social relationships with one another, including identifying an exercise partner. Additionally, to connect participants to a medical home, physicians and nurses at Eau Claire Cooperative Health Centers—IAW healthcare partner—hosted a meeting for participants at their health center.

CHWs delivered the project activities in 7-week sessions in community settings to groups of 20 to 33 participants. The intervention was conducted in two cycles, Cycle 1 and Cycle 2 (Class I and Class II); Cycle 2 classes were taught concurrently. Each cycle/class of participants received the same 7-week session and participated in auxiliary activities. Cycle 1 was conducted with residents from the subsidized housing community in Richland County, while Cycle 2 consisted of women from other communities in Richland County.

### 2.6. Data Analysis

Descriptive statistics were used to analyze all data for this study. For individual program participants, differences between pre- and postintervention health outcomes (e.g., weight, glucose levels, body mass index levels, blood pressure, and cholesterol), knowledge of approaches for prevention of obesity and diabetes relative to the IAW curriculum sessions, levels of exercise and engagement in fitness activities, enrollment in health insurance plans, and visits to primary care doctors were examined. Relative to programmatic areas, several key outcomes to measure the effectiveness of the program were evaluated. An analysis of cohort level outcomes did not demonstrate a statically significant variation in outcomes between Cohort I (public housing residents) and Cohort II (nonpublic housing residents). In depth analysis of outcomes by cohort did not display statistically significant variations in postintervention measures of increase in knowledge between public housing residents (Cycle I) and nonpublic housing residents (Cycle II). As residents resided within the same zip code with homogenous background and health factors the two groups' results were combined.

## 3. Results and Discussion

### 3.1. Background/Demographic Information

**Figure 1:**
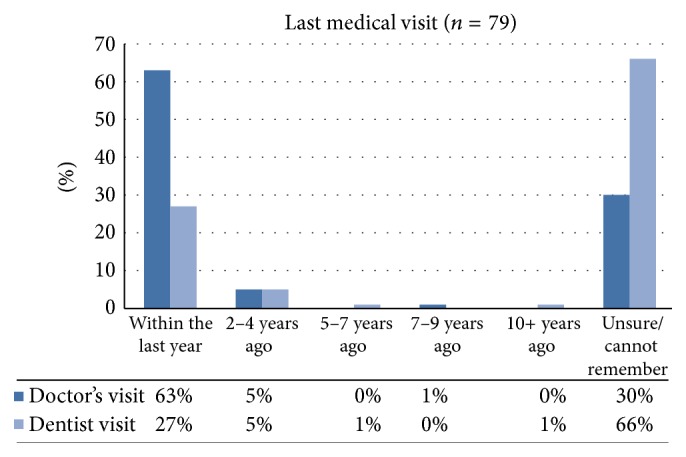
Intervention healthcare access and utilization.

Overall, the median age of participants was 29 years, with a range of 18 to 74 years. Most participants were not married and had education beyond high school. Approximately the same proportion of participants was employed (28%) as compared to unemployed (27%). While the median annual income for participants was $15,000 per year, the majority (71%) had an annual income of $15,000 to $25,000. When asked if they had ever been diagnosed with a number of health conditions, 43% of the women reported that they had been diagnosed with nutrition-related diseases (13% diabetes, 4% cancer, 25% cardiovascular disease (including high blood pressure), and 1% eating disorder). Regarding healthcare access and utilization, almost half (46%) of participants and their family members (44%) were uninsured. The majority of participants (65%) reported having a primary care physician; however, most who reported seeing a physician in the last year (63%) went to hospital emergency rooms, not their primary care physician, even though the majority of the reasons for the visits were nonemergency primary care in nature. Almost third of participants (30%) were unsure of or could not remember when they last visited a physician (Figure 1).

### 3.2. Exercise Habits

**Figure 2:**
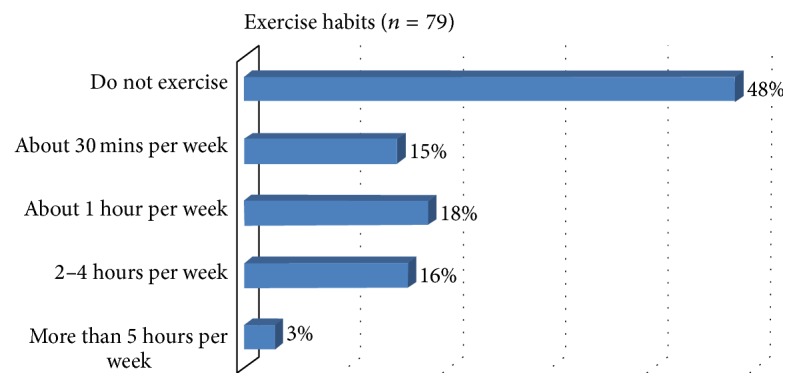
Preintervention exercise habits.

When asked if they currently exercise, 48% of women responded “no.” The remaining 52% reported usually exercising ≤1 hour (33%), about 2–4 hours (16%), and >4 hours (3%) a week (Figure 2). Participants who did not exercise identified several barriers to exercising including lack of places to work out, limited time to work out, financial barriers, safety issues, lack of interest, difficulties due to work and family responsibilities, and boredom. The majority of women reported several of these issues as obstacles to engaging in a regular routine of physical activity and/or exercise.

### 3.3. Preintervention (Baseline) and Postintervention Outcomes

**Figure 3:**
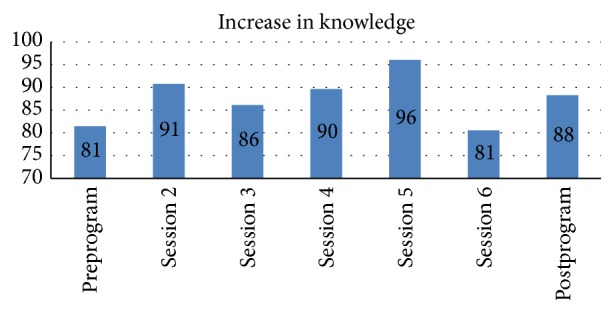
Postintervention knowledge assessment.

One of the primary outcomes of interest was participants' knowledge about nutrition and its impact on health. A seven-point score improvement was realized across the five nutrition topic areas from pre- to postintervention levels on average. Based on a 100-point scale, the average preprogram score was 81, and the average postprogram score was 88. After intervention, participants were most knowledgeable about the topic of Strategies for healthy eating and exercise and least knowledgeable about partnering with your healthcare provider concepts, with scores ranging between 81 and 96 points. This suggests moderate increases in levels of knowledge among program participants relative to the domains of interest (Figure 3).

### 3.4. Health Screening and Vital Statistics

#### 3.4.1. Blood Pressure, Blood Sugar, and Blood Cholesterol

Blood pressure, blood sugar, and cholesterol values were screening measurements and were not paired with classifications of hypertension, diabetes, and cholesterol. Instead, the terms normal, borderline/borderline high, and high were used to describe the values. Pre/postresults suggested that there was a decrease in blood pressure and blood glucose levels. Participants with high vital statistics before intervention in relation to high blood pressure and blood glucose levels demonstrated borderline values by the end of the program, thus indicating improvement in health measures. However, cholesterol levels remained only marginally changed indicating a longer duration between cholesterol testing with increased focus on cholesterol lowering tactics being necessary for future interventions. In terms of the nonfasting blood glucose levels, 85% of participants prior to the intervention compared to 94% after the intervention had a normal level (70–139 mg), a 9% increase in the prevalence of normal blood glucose. At baseline, 26% of participants had normal blood pressure (<120 and <80 mm Hg) and 43% had a high reading (≥140 or ≥90 mm Hg), compared to 28% and 53%, respectively, after the intervention. The utilized classification was defined by the American Heart Association [19]. The cholesterol levels of women remained relatively the same (Table 1).

#### 3.4.2. Weight and Body Mass Index

Participants lost an average of approximately one kilogram each, increasing the percent of participants who had a normal body mass index (BMI) of 18.5 to 24.9 kg/m^2^ preintervention (8%) to 9% after the intervention. BMI, which is weight in kilograms divided by height in meters squared kg/m^2^ (rounded to the nearest tenth), was calculated based on participants' measured heights and weights. Prior to the intervention, over three-fourths of participants (78%) had a BMI of ≥ 30.0 kg/m^2^, with 21% of those having a BMI of ≥ 40.0 kg/m^2^. The former BMI is within the range of obesity and the latter one indicates extreme obesity, as defined by the National Institutes of Health (NIH) [20]. After intervention, the prevalence of obesity and extreme obesity combined decreased to 76%, with no change in the prevalence of extreme obesity. None of the participants were underweight (BMI of < 18.5 kg/m^2^) before or after the intervention.

#### 3.4.3. Connection to a Primary Care Home

Several participants enrolled in services at the Eau Claire Cooperative Health Centers, which resulted in 14% of participants establishing a medical home and 31% seeing a primary care physician. In addition to this healthcare access outcome, three participants enrolled in general educational development (GED) classes and one received her GED certificate as a direct result of her involvement in the project.

## 4. Discussion

This project shows that a culturally tailored and gender-specific intervention complemented with exercise sessions (1) increased participant knowledge related to the complex of nutrition, physical activity, and health; (2) improved select screening health indicator values; and (3) increased the proportion of participants with a routine source and location of healthcare, including a medical home. These preliminary findings are important because they add diversity and depth to the body of scientific literature examining community-based, collaborative interventions that may help to reduce the nutrition-related chronic disease burden among African Americans; particularly among disadvantaged African American women.

The mean baseline knowledge level about nutrition, physical activity, and diet-disease relationships was satisfactory before the intervention (an average preintervention assessment score of 81/100) but still substantially improved after the intervention (an average postintervention assessment score of 96/100). Because most participants in the project were the primary food purchasers and preparers, as well as the heads of their households, their nutrition knowledge significantly impacts the quality of their families' nutrition. In America, mothers customarily educate children about food and nutrition [21, 22], and women typically assume the lead role in food shopping and preparation in most families. Therefore, women are most likely to make food and diet choices for the family [21]. Research demonstrates that knowledge of nutrition, health, and improvement in healthy food preparation play an important role in socioeconomically disadvantaged women providing food for their families. Thus, these factors impact families' decisions to adopt healthful dietary behavior and women's abilities to serve as positive role models for their children. The role of the female head of household in preventing and managing overweight/obesity is critical to the health of children and future generations [23]. Several researchers have examined the influence of parents on the eating patterns and weight of children and corroborate the significant role of the mother in children's health-related decisions [24, 25].

The vast majority of IAW participants were overweight (15%) or obese (78%), with approximately a fifth being extremely obese. An overwhelming majority of participants did not exercise or exercised much less than recommended by the Federal Physical Activity Guidelines prior to the intervention [15]. Overweight, obesity, and limited exercise increase the risk for several diet-related health conditions and negatively impact the control of these conditions. Overall, this study's findings are consistent with data from previous studies. One study found that over three-fourths of its nonrandom sample of low-income women were also overweight or obese [23]. National data revealed that only 11% of African American women, compared to 19% of white American women, meet the Federal Physical Activity Guidelines [15] and similar to this study's findings, over half (52.6%) of the African American women, excluding workplace physical activity, were physically inactive [26]. The striking inadequate physical activity among participants at baseline coupled with excess weight suggests that physical inactivity, in addition to poor nutrition, may be a critical area to target in prevention interventions.

The high screening blood pressure among almost three-fourths of participants suggests that a high prevalence of uncontrolled hypertension among participants may exist. Nationally, 43% of African American women in 2005–2008 had hypertension, compared to only 28% of non-Hispanic white women and 25% of Mexican women [15], but the blood pressure of only 34% of African American adults with hypertension is under control [27]. Uncontrolled blood pressure is a serious public health problem among African Americans and should be aggressively targeted through long-term nutrition and physical activity interventions in conjunction with appropriate clinical treatment.

## 5. Limitations

The findings in this study are subject to six primary limitations. First, the project participants were self-selected, primarily obese, socioeconomically disadvantaged African American female population in the Southeastern United States. The generalizability of the findings may, therefore, be limited. Second, most of IAW data are based on self-reported information and are subject to recall errors and response biases (e.g., over- or underreporting of actual exercise [28]). Third, a random blood glucose test and a total cholesterol test without a profile were performed because of participant barriers and limited resources, respectively. Fasting blood glucose and cholesterol profile results would have been more clinically meaningful. Fourth, the short duration of the IAW Program limits our ability to determine the positive long-term health implications for program participants. This limitation is noticeable with the increased number of participants with hypertensive vital measures after intervention. The duration of the intervention was limited due to funding restrictions. Additionally, while it would have been ideal to conduct a postintervention analysis at a time further away from the last program session, the preliminary postprogram results are indicative of improved health status among the vast majority of program participants. Because CHWs were engaged in the IAW Program and were provided with training on how to conduct IAW sessions, community laypersons are uniquely positioned to continue health education among program participants and, therefore, have long-term impacts on participants' health. Future studies will conduct longitudinal research on women who participated in more than one cycle of the IAW Program so that the long-term impacts on vital statistics may be ascertained. For the purposes of study replication, we recommend a longer duration of the intervention as well as prolonged follow-up.

Fifth, there was a limited difference between pre- and postintervention knowledge. Moreover, Session 6 demonstrated a significant decrease in knowledge. In our analysis, we found that the decrease in the average score is attributed to participants' understanding of Question number 1 on Session 6 after test. The question reads, “Who would make up your healthcare team?” The options available were (a) Only a Primary Physician; (b) Family and Friends; (c) Primary Physician, Nurse, Dietician, Dentist; (d) Both A and B. Participant responses likely varied due to the session content and description of a comprehensive healthcare team, which included various healthcare professionals and family/friends to support and assist with routine primary care visits. The correct answer for the session test identified the key health professionals that should be included in an individual's healthcare team but was not inclusive of positive social support (i.e., friends or family) as promoted in the curriculum to assist in maintaining an optimal health status. Lastly, program participants were provided with incentives to participate in the IAW Program. Although the participants were of low socioeconomic status, the incentives did not significantly impact postintervention results as the average incentive received per participant was 10–25 USD—with 20 USD being the average—for the 3-month program. Participants were given an incentive (10 USD) for attending the educational portion of the program and were given 10 USD for completing the program. The incentives were provided infrequently and were primarily used to obtain the initial interest of program participants. Despite these inherent limitations, the study yields important implications for the overall health improvement among African American women and an intervention model that can facilitate these improvements.

## 6. Conclusions

While socioeconomic and physical environments can affect opportunities for healthy behavior, the culture, health-related knowledge, and resource-related limitations of communities must also be addressed when developing interventions. The IAW intervention utilized a community engagement research methodology in the design, implementation, and evaluation of the intervention. Because of this community-based participatory approach, participants have the opportunity to sustain and continue to benefit from program results. The CHWs who facilitated program sessions reside within the same communities as the intervention participants and are in a unique position to continue disseminating health-related information to community residents. Through the IAW Program, CHWs have also been trained in assisting community residents in establishing and maintaining a primary care home and are equipped to follow up with IAW participants, ensuring that optimal patient-provider communication is maintained. In these ways, the IAW Program establishes sustainability as well as capacity building in an effort to address the prevalence of obesity, diabetes, and associated chronic sequelae even after the intervention concludes. The outcome of our efforts, related to a familiar program facilitator and a familiar location of healthcare, suggests that a community-based participatory intervention can have a substantial positive impact on access to healthcare for certain populations. Our preintervention data may indicate that many participants have been using emergency rooms as their usual facility for care. African Americans are almost twice as likely as whites to visit the emergency department for primary care conditions [1]. Individuals who do not have access to a convenient routine source of primary preventive healthcare are more likely to visit the emergency department or be admitted to the hospital [3]. Our intervention results convey that access to a convenient source of primary care may result in considerably improved health outcomes and may help to reduce or eliminate health disparities [3, 29, 30], regardless of income or insurance status.

The primary practical implications of IAW are twofold. First, to prevent, delay the onset of, and control chronic diseases through improved nutrition and increased physical activity, there must be a combined capacity and efforts of communities, healthcare professionals, voluntary and professional organizations, the private sector, governmental agencies, policymakers, and academic institutions. CHWs, particularly in disadvantaged communities, can play a vital role in community-based initiatives, as demonstrated in IAW. Additionally, programs and policies should reflect the critical roles women and parents play in lifestyle behavior of families. Second, policy and environmental changes that can promote evidence-based social and systems approaches that support proper nutrition and adequate physical activity for individuals, families, and the communities must be made [5]. For example, food and beverage companies should meet both the health and nutritional needs of consumers. Toward this end, more emphasis should be placed upon increasing the marketing of healthy foods (including snacks and drinks for children); using standardized transparent food labels; and providing foods lower in saturated fats, trans fats, cholesterol, sodium, and added sugars.

To advance health equity, future studies may expand on this promising health education model by including specific details of dietary and food purchasing and preparation patterns among a larger population. With the primary influence of African American women in their households, the implementations of interventions similar to IAW have the capacity to support the attainment of health equity among high-risk women, their families, and communities. Additionally, longitudinal research should be conducted to examine whether knowledge and skills gained in such an intervention continue to affect purchasing, dietary, and physical activity, as well as healthcare utilization in the future. Changing these behavior requires an ongoing, strong commitment to be sustained and effective. The IAW Program serves as a community-based participatory model that facilitates the establishment and maintenance of such health-related behavioral changes among African American women.

## Figures and Tables

**Table 1:** Preintervention and postintervention measured screening health indicators (*n* = 79).

Measured indicator	Preintervention, %	Postintervention, %	Change%
Blood pressure (mm Hg)—systolic and/or diastolic			
<120 and <80 normal	26	28	+2
120–139 or 80–89 borderline	30	19	−11
140–159 or 90–99 high	17	25	+8
≥160 or ≥100 high	26	28	+2

Blood glucose, random (mg/dL)			
70–139 normal	85	94	+9
140–199 borderline	10	0	−10
≥200 mg high	6	6	0

Total blood cholesterol, random (mg/dL)			
<200 desirable range	82	83	+1
200–239 borderline high	12	11	−1
≥240 high	6	6	0
